# Editorial: Plant Root Interaction With Associated Microbiomes to Improve Plant Resiliency and Crop Biodiversity

**DOI:** 10.3389/fpls.2021.715676

**Published:** 2021-07-02

**Authors:** Nikolay Vassilev, Eligio Malusà, Davide Neri, Xiangming Xu

**Affiliations:** ^1^University of Granada, Granada, Spain; ^2^Department of Plant Protection, The National Institute of Horticultural Research, Skierniewice, Poland; ^3^Marche Polytechnic University, Ancona, Italy; ^4^NIAB EMR, Department of Pest and Pathogen Ecology, West Malling, United Kingdom

**Keywords:** plant-associated microbiome, biocontrol, biofertilization, soil, agronomic strategies

Plant beneficial microorganisms are now accepted as potential alternatives to chemical fertilizers and pesticides in agriculture. However, despite the enormous research efforts, there is still much to be learnt about the underlying processes that affect the efficacy of biocontrol and biofertilizer products in crop systems. A deeper ecological understanding of complex interactions among introduced beneficial strains and resident microbiota under changing environment is essential (Xu and Jeger, 2020). These complex interactions are greatly influenced by the formulation of beneficial microbial strains (Vassilev and de Oliveira Mendes, 2018) as well as the growing substrate characteristics (Ponzio et al., 2013) and commercial agricultural practices (Malusá et al., 2016). This Research Topic presents 33 articles that describe the recent research progresses on the exploitation of beneficial microbes in agriculture.

**The influence of management practices** on plant-associated and soil microbiome was explored on a number of crop species, including pea and tomato. Accumulation of several fungal root pathogens, reduction of specific potential beneficial bacteria, and gradual decrease of soil pH value in soils are identified as possible causes contributing to reduced productivities of American ginseng associated with continuous monocropping (Zhang et al.). Removal of pruned branches from tea orchards can prevent accumulation of allelochemicals and avoid soil health issues in monocropping through the changes in the soil microbiota as influenced by pruning wastes on the orchard ground (Arafat et al.). Fungal communities in growing substrate were greatly affected by crop rotation of tomato with cabbage, bean, or celery in greenhouse (Lyu et al.). Soil management, crop residue management and tillage treatments all affected the bacterial community structure in both bulk soil and pea rhizosphere, but the plant itself was the dominating factor shaping the bacterial communities (Chaudhari et al.).

**Plant genotypes** have the ability of driving the composition of microbial species in their rhizosphere. Thus, different blueberry species, although sharing a common core rhizobiome, considerably shaped rhizosphere community structures of both prokaryotic and eukaryotic microorganisms (Li et al.). In addition to specific genotypes, phenotypes can also be associated with phytobiomes. For instance, large differences in cotton rhizosphere microorganisms and root endophytes were associated with resistance to *Verticillium dahliae* (Wei et al.). The large differences in the rhizosphere fungal community structure were related to the differing tolerance of soybean genotypes to aluminum (Shi et al.). Thus, identifying the genes regulating host/microbe associations/interactions is expected to provide valuable targets for breeding new varieties capable of assembling a healthier microbiota (Corbin et al.).

The impact of **abiotic factors** on phytobiome was demonstrated for diverse environments. Karray et al. showed the prominence of the climatic aridity gradient on the microbial community composition in the rhizosphere and endosphere of prickly pear, a typical crop of arid environments. Temperature significantly affected the transmission frequency, mycelial biomass and alkaloid production of an endophyte (*Epichloë* sp.) that was isolated from a wild grass and inoculated into perennial ryegrass to improve its resistance to herbivores (Freitas et al.). The molecular mechanisms underpinning the ability of an actinobacterial strain (*Microbacterium* sp.) to improve pepper plant tolerance to water stress were related to the differential expression of osmoprotectant producing proteins, reactive oxygen species scavengers, plant-hormones, structural proteins and signaling proteins (García-Fontana et al.). Moist conditions and the presence of an endophyte (*E. gansuensis*) influenced both root-associated and rhizospheric soil bacteria communities of a needlegrass plant (*Achnatherum inebriens)* (Ju et al.). Dark septate endophytes (DSE) and their habitat of origin were associated with the plant response to drought conditions (He et al.). Otlewska et al. reviewed the microbial ecology and interactions between endophytic communities and host plants occurring in saline agricultural soils and discussed on the potential of using PGPR to increase plant's salt stress tolerance. Such an approach was demonstrated to be useful in wheat where inoculation of wheat plants with a nitrogen-fixing *E. cloacae* HG-1 strain not only resulted in increased plant growth and salt stress tolerance, but also affected microbial community structure (Ji et al.).

Plants are often symbiotically related with beneficial microorganisms in soils often poor in nutrients. However, the genetic and molecular phenomena behind such symbiotic relationships are still unclear. Several articles dealt specifically with the **nitrogen fixation**. Both the soybean nodulation phenotype and the nitrogen level affected the rhizosphere bacteria community, but the nodulation phenotype contributed more than the N-supply (Wang et al.). Using a *GmcA* mutant strain of *Rhizobium leguminosarum* with a decreased antioxidative capacity, Zou et al. showed that GmcA oxidoreductase is crucial to both the establishment of the symbiosis and the nitrogen fixation capacity. Several nuclear genes and the symbiotic plasmid gene present in *Bradyrhizobium* strains also affected the symbiosis incompatibility found for Type II strains in comparison to Type I strains when a host plant not belonging to the common host species was inoculated (Wu et al.).

Several articles dealt with the exploitation of soil microorganisms for **biocontrol of plant diseases**. Exploiting compost microbiomes for biocontrol of soil-borne diseases through isolation of biocontrol and PGPR strains and functional assays with genomics approaches were used as tools to improve the biocontrol efficacy of composts (Lutz et al.). The suppressiveness of compost was, indeed, largely influenced by its microbiota composition and the applied dosage, as shown in the *Phytophtora capsici*—*Cucurbita pepo* pathosystem (Bellini et al.); furthermore the ability to shape microbial composition in the rhizosphere appeared to be an important attributes of suppressive composts. An inundate introduction of a single *Pseudomonas chlororaphis* strain could also increase relative abundance of few prokaryotic families in the soil, leading to improved biocontrol of *R. necatrix* in avocado (Tienda et al.).

Plant roots exudates and particularly allelochemicals are believed to have played significant roles on protecting plants from biotic and abiotic stresses through their influences on soil microbial communities. A higher concentration of phenolic acid exudated from rice roots, due to overexpression of one gene from the *PAL* family, resulted in an increased population of *Myxococcus* sp. in the rhizosphere soil and led to improved allelopathic inhibition of weeds (Li et al.). Similarly, the types and concentrations of phenolic acid root exudates in a maize-soybean intercropping system were crucially important for suppression of *Phytophthora* diseases (Zhang et al.). On the other hand, biocontrol efficacy could be reduced due to the interaction of biocontrol strains with resident microbiota. For instance, quorum sensing disruption mediated by specific root exudates and by an increased quorum quenching bacterial community may have contributed to reduced antagonistic effects of *Pseudomonas* spp., as observed in the *Rehmannia glutinosa* rhizosphere under long-term monoculture (Li et al.). Interestingly, plant growth promoting effects observed after application of only the fermentation broth of a beneficial microbe was related to a modification of the rhizosphere bacterial community (Lu et al.).

One current research focus is to better understand the biochemical and molecular mechanisms involved in plant–microbe–soil interactions and the **multifunctionality of soil microorganisms**. Razinger et al. reported multifaceted functions (i.e., plant protection and growth promotion) of entomopathogenic fungi, but the observed effects depended on either specific fungal strains, plant species or genotypes. One difficulty in exploiting multifunctional strains in commercial agriculture is, however, related to regulatory hurdles, which may vary greatly among regions (Kowalska et al.). These regulatory difficulties may significantly impair a wider use of beneficial microorganisms despite our improved understanding of their interactions with plants and native microbiota as well as their specific pre-, pro-, and post-biotic functions (Vassileva et al.). DNA-based techniques may, however, support the regulatory process specifically in the detection and quantification of detrimental effects on soil microorganisms and non-target organisms at sub-lethal doses (Vischetti et al.), which is a subject under consideration by regulators world-wide in view of the UN Sustainable Development Goals. Moreover, Qui et al. demonstrated that the DNA-based analysis of fungal/bacterial community composition was robust to preservation methods of plant samples and the primer sets used, suggesting that simultaneous analyses of plant and soil microbiomes are possible.

**Production, formulation and application of biofertilizers** are the final step for exploitation of specific beneficial strains in agriculture. Encapsulation technology for microorganisms may provide additional benefits for formulations containing different additives, including phyto-stimulants (Vassilev et al.). The opportunities and bottlenecks for new bioproducts based on endophytic microorganisms (Chitnis et al.) or on synthetic microbial consortia tailored with microorganisms possessing traits for robust colonization, long persistence and specific beneficial functions (de Souza et al.) were reviewed, particularly in terms of mitigating abiotic and biotic stresses.

In conclusion, the aim of this Research Topic was to improve our knowledge on processes and their biological mechanisms that affect the efficacy of biocontrol and biofertilization in crop systems. These included plant genotypic effects, rhizosphere and soil microbiome composition, endophytes, formulation, and production of bioinocula and commercial agronomic practices. The articles included in this e-book are addressing all these aspects, pointing to new research areas that can improve our understanding of the mechanisms of plant-microorganisms interactions in order to support and expand practical application of bioinocula in agriculture, which will reduce the negative environmental and social impact of synthetic chemicals in agriculture, and support the new Green Revolution we all aspire to.

## Author Contributions

All authors listed have made a substantial, direct and intellectual contribution to the work, and approved it for publication.

## Conflict of Interest

The authors declare that the research was conducted in the absence of any commercial or financial relationships that could be construed as a potential conflict of interest.
